# Impacts of small-scale irrigation on farmers' livelihood: Evidence from the drought prone areas of upper Awash sub-basin, Ethiopia

**DOI:** 10.1016/j.heliyon.2023.e16354

**Published:** 2023-05-16

**Authors:** Husen Maru, Amare Haileslassie, Tesfaye Zeleke

**Affiliations:** a Center for Environment and Development Studies, Addis Ababa University, P.O. Box 1176, Addis Ababa, Ethiopia; b Department of Geography and Environmental Studies, Wolaita Sodo University, P.O. Box 138, Sodo, Ethiopia; c International Water Management Institute (IWMI), P.O. Box 5689, Addis Ababa, Ethiopia

**Keywords:** Small-scale irrigation, Livelihood capital assets, Propensity score matching, Drought-prone areas, Upper awash sub-basin, Ethiopia

## Abstract

Irrigation is an important mechanism to mitigate risks associated with the variability in rainfall for the smallholder subsistence farming system. This study analyzed how practicing small-scale irrigation (SSI) impacts the key livelihood assets on farm households' human, physical, natural, financial, and social capitals in Ethiopia's upper Awash sub-basin. The household-level survey data, collected from the 396 sample households, was used to carry out the current study. A Propensity Score Matching (PSM) analytical model was applied to match the SSI user and non-user groups. The difference between the five capital assets of livelihood was estimated using the PSM's Nearest Neighbor, Radius, Kernel Mahalanobis, and Stratification matching criteria. The results indicated that farmers' participation in SSI has enhanced the capital assets of the farm households. Compared to the non-users, the irrigation users were better off in the number variety of food consumed (0.28 ± 0.13 Standard Error [SE]), types of crops produced (0.60 ± 0.17 SE), expenditures on land renting, and agricultural inputs (3118 ± 877 SE) measured in Ethiopian Birr (ETB), as well as on-farm (9024 ± 2267 SE ETB) and non-farm (3766 ± 1466 SE ETB) incomes. Challenges such as the involvement of local brokers in the market value chain and the absence of farmers' marketing cooperatives have reduced the benefit of irrigated agriculture. Hence, the expansion of SSI schemes for the non-user farmers should consider improving the water usage mechanism and productivity, establishing proper water allocation institutions between up and down streams and limiting the role of brokers in the irrigation product marketing chain be future policy directions.

## 1 Introduction

Small-scale irrigation has enhanced agricultural production by increasing cropping frequencies and production, though the magnitude of this effect varies significantly across the globe [1,2]. The livelihood of farmers using irrigation is more resilient than that of the rainfed-dependent farmers globally [[3], [4]]. This is because, compared to rainfed agriculture, irrigation has proven to be a better alternative for increasing the availability and quality of water resources for farming [5], thereby contributing to agricultural sustainability and livelihood improvements.

In Africa, irrigation has a more significant potential to supplement the rainfed agricultural production system and improve the life of smallholder farmers [6,7]. It is important to expand irrigation in Africa, especially in Sub-Saharan Africa, to support farmers' highly vulnerable livelihood and their endeavors out of poverty [8,9]. However, the expansion of irrigation needs a timely inspection of its role in improving the farmers' livelihood [10].

Agriculture continued to be Ethiopia's most important economic sector, as its development heavily depended on it [[11], [12], [13]]. However, agricultural activities in Ethiopia are predominantly dependent on rainfall [14,15] which is dominantly practiced by smallholders, making the country's food production system very vulnerable to climatic variability and extreme events like drought [16,17]. Variations in rainfall amount, distribution, trends, and rising temperatures directly impacted rainfed agriculture productivity and farmers' livelihoods who rely heavily on agriculture [[18], [19], [20]]. In drought-prone parts of Ethiopia, production from rainfed agriculture is highly variable [[21], [22], [23]], corresponding to the amount, the duration, and the spatial distribution of rainfall [24]. To tackle the impact of the rainfall variability in the areas of abundant surface water, supplementing rainfed agriculture with small-scale irrigation-based schemes is encouraged by the government of Ethiopia [25]. Maru et al. [26,27] also proved that irrigation is essential for building farmers' resilience to drought.

Small-scale irrigation scheme takes place on plots of land (5 to 200 ha) [28], where individual farmers have the bulk of control and use technologies that they can run and maintain [29,30]. The individual farmer's plot of land varies between 0.25 ha and 0.5 ha [31]. According to Passarelli et al. [32], more focus should be on Ethiopia's small-scale irrigation. This is because the dominant farmers in the country are smallholders with limited farmland size. Smallholder farmers can plan and manage it at the community level (farmer-led irrigation). Moreover, small-scale water supply schemes need only involve the *Kebele* administration and the local community [33]. *Kebele* is the lowest administrative structure in Ethiopia. Thus, decentralizing water resources development means that local authorities, the *Kebele,* and community administrations, should play a more active role in planning and implementation [34].

The current study was conducted in the upper Awash sub-basin (UASB). Awash Basin is Ethiopia's most utilized river basin [35,36]. Households in the Awash Basin are known for their subsistence mixed-crop-livestock production system [26]. Most rely on the rainfed system and naturally available moisture for agriculture [27,37]. Upper Awash is distinguished from the middle and lower parts due to the relatively high concentration of rainfed and small-scale irrigation agricultural practices [38,39]. The livelihood of farmers in the middle and lower parts of the basin is mainly pastoralism.

Previous studies indicated that small-scale irrigation has improved crop production and enhanced overall rural livelihoods [26,[40], [41], [42]]. However, in these studies, there are a lot of generalizations. There was no explicit evidence that supported how irrigation impacts the livelihood capital of the irrigators. Because rural livelihoods are heterogeneous, generalizations and aggregations on the effects of SSI on rural livelihoods are misleading [43].

Studies on irrigation and livelihood were carried out in various parts of Ethiopia. The dominant irrigation practices have been in northern Ethiopia (e.g., Refs. [[44], [45], [46], [47]]). The main focus of these studies was analyzing the role of small-scale irrigation on the livelihood of the farm households taking a single or a few livelihood indicators such as income, food security, and poverty. Studies are conducted on irrigation and livelihood improvements in other locations in Ethiopia. To mention some Southern Ethiopia [48,49], Eastern Ethiopia [33], and Western Ethiopia [50]. A generalized approach, such as representing livelihood with a single indicator, was also the focus of these studies. Methodological gaps have been observed in some related studies as some used descriptive statistics, such as percentages and frequencies, to measure the impacts of SSI on livelihood (for example, [44]). Due to the irrigation intervention, this methodology could hide the actual difference in livelihood capital assets between SSI irrigation users and non-users. This can be best captured by model-based analysis methods such as PSM. PSM method was used by various studies in Ethiopia and elsewhere to analyze the impact of small-scale irrigation on livelihood components (for example, [ [[51], [52], [53]]).

In studying the impacts of irrigation on the livelihood improvement of farmers, it is essential to consider factors that affect farmers' livelihoods and the relationship between them [54]. Furthermore, the study must focus on what aspects of their livelihood are improved by irrigation and the trade-offs between those aspects [[55], [56]]. This information is also crucial for development practitioners, policy formulators, and decision-makers to expand SSI based on their essential role in improving the farmers' livelihood.

Upper Awash sub-basin is one of the drought-prone areas of Ethiopia, where smallholder farmers suffer continuous crop production reduction, crop failure, and livestock deaths due to moisture stress [26,27,57]. This is because most farmers use the naturally available rainfall for their agriculture, which is highly variable at an interannual and intra-annual scale [35]. Hence, irrigation is being expanded in the sub-basin to reduce moisture stress challenges on crops and livestock, improving smallholder farmers' livelihoods. Farmers' participation in small-scale irrigation is expected to improve their livelihoods, and assessing the impact of irrigation on livelihood capitals is timely.

The main source of irrigation in the study area is river water through SSI schemes. The Water Users' Association (WUA) does the irrigation water allocation. Farmers use open channel gravity to reach the water to their farmlands. Since the government implements the SSI structures, the major costs associated with irrigation practice are the costs of land, agriculture inputs, and labor. During malfunctioning, the farmers maintain the SSI structures unless the cost is huge. When a large maintenance cost is required, the government handles it.

This study aims to analyze the impact of SSI on smallholders' livelihood improvements, taking into account the five capitals (human, physical, natural, financial, and social) through quantitative data (through the PSM model) and qualitative data. The study's outcomes can inform the government and irrigation development practitioners to identify the livelihood capitals enhanced by small-scale irrigation usage.

## 2 Materials and methods

### 2.1 The study area-location and characterization

#### 2.1.1 Location and characterization of the study area

**Fig. 1:**
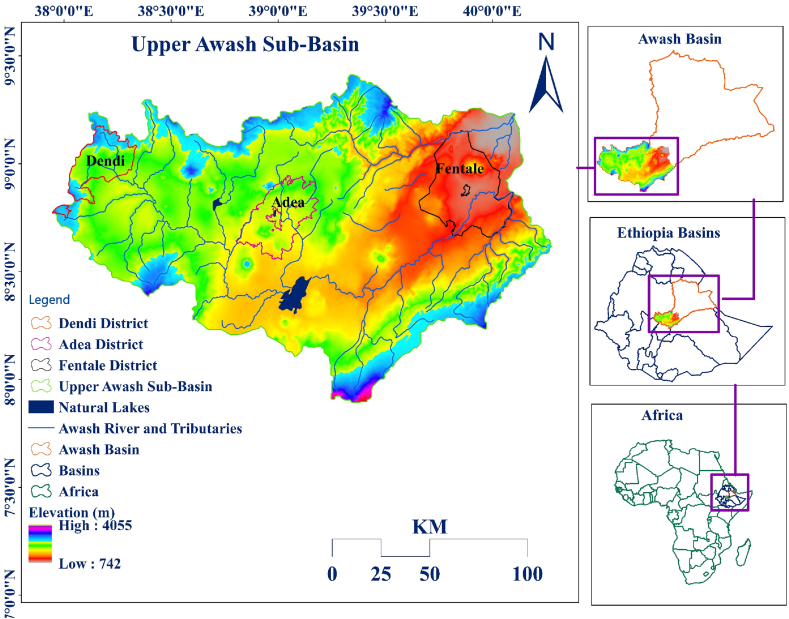
Location map of the study area showing elevation and sample districts.

Awash is one of the 12 river basins in Ethiopia. Based on physical, climatological, agricultural, social, and water resource characteristics, the basin is divided into three parts: lower, middle, and upper [58]. The current study's geographical scope is the basin's upper part (Fig. 1). Geographically 37°54′35″ E−40°16′53″ E longitude and 7°53′15″ N–9°25′15″ N latitude limits define the study areas, which cover 24,545 square kilometers. Administratively the study area encompasses three districts: Dendi from the highland, Adea from the midland, and Fentale from the lowland agro-ecological zones.

The climate condition of the upper Awash sub-basin ranges from arid to afro-alpine. The annual temperatures average between 15 and 20° Celsius. In the upper Awash sub-basin, altitude ranges from 4055 to 742 m above mean sea level (AMSL) (Fig. 1), implying a wide altitude range within the study area [59], which influences local biophysical settings and associated livelihood activities (e.g., climatic factors). The mean annual precipitation in the sub-basins ranges from 800 to 1400 mm, depending on the elevation difference [60]. The duration and amount of precipitation show variations in the different seasons. The primary rainy season, known in Ethiopia as *Kiremt*, lasts from June through August. The movement of the global atmospheric system called the Inter-Tropical Convergence Zone (ITCZ) to the Northern hemisphere brings a large amount of rainfall in Ethiopia and the sub-basin during this season. Plantation of the most rainfed crops occurs during the rainy season, and the need for irrigation water is less.

The sub-basin is very dry during the Ethiopian *Bega* season (December to February), and irrigation is vital. The temperature is high during this season, so high evapotranspiration is experienced. During the *Tsedey* season (September to November) and *Belg* season (March to May), there is some rainfall in the sub-basin. Still, it cannot support crop and pasture production unless it is supported by irrigation water.

#### 2.1.2 Livelihood characteristics of study farmers

Farmers' livelihoods in the highlands and midlands rely on crop-livestock mixed systems that take advantage of naturally occurring rains. Wheat, teff, barley, maize, peas, beans, enset, and potatoes are among the dominant crops grown in the highlands (e.g., Dendi District) [61]. Maize, wheat, teff, and other cereals are the most important crops in the Adea District (midland). In the midland and highland areas, livestock is incorporated into crop production and is a source of income while enhancing the crop production system through using manure as fertilizer. Crops and livestock are complementary components, with livestock supporting crop production via animal plowing and agricultural leftovers feeding animals [62,63]. Lowland areas are known for their agropastoralism and pastoralism [64]. Sorghum and maize are common crops. In lowlands, households' significant assets depend on animals and their products. In Fentale, goats, camels, and cattle are the most common livestock.

The reduction amount and seasonal variability of rainfall necessitate the shift from rainfed to irrigation agriculture to sustain the most fragile livelihood of the farmers. There are two types of farmers in the study sub-basin. The first is only rainfed dependent who produce crops and livestock based on rainy seasons (*Kiremt* and *Belg*). During the non-farm periods, some engage in trade and daily labor to support their livelihood. They are non-irrigators mainly because they do not have irrigation land or good water access.

The second category of farmers is irrigators. They produce crops using irrigation during the dry season and a rain-fed system during the rainy season. The probability of drought-induced crop failure and production reduction is less than that of rainfed farmers. This category of farmers has better crop production and diversified income sources. Sometimes, they hire labor for their irrigation activity [26].

### 2.2 Sampling and data sources

#### 2.2.1 Sampling

There are three major agro-ecological zones (AEZs) in the upper Awash sub-basin: highland, midland, and lowland. The target sample households were chosen using a three-stage sampling approach. First, Dendi District was selected to represent the highland, Adea District to represent the midland, and Fenatle District to represent the lowland AEZs. Second, the sample size was calculated using Equation (1) [65]. This formula was preferred because it provides an acceptable sample size, important in any survey requiring the optimum number of participants to generate ethically and scientifically valid results.

(1) $$n=\frac{2\left(Z_{\alpha }+Z_{1-\beta }\right){2\sigma }^{2}}{\Delta^{2}}$$

where

n=RequiredsamplesizefortheHHsurvey;

$Z_{\alpha }=Constant\;\left(1.96\right)$ at 5% margin of error;

$Z_{1-\beta }=Constant\;\left(1.6449\right)$ at 5% margin of error;

σ= the standard deviation (estimated);

Δ=estimatedeffectsize.

Equation (1) suggested a total sample size of 360 households. The total sample size is 396 (198 irrigators and 198 non-irrigators) after accounting for a 10% non-response rate (36 people). Thirdly, respondent household heads were chosen at random using a lottery method. Two Kebeles (the lower administration structure in Ethiopia) from every three districts were chosen based on both availabilities of irrigator and non-irrigator farm households in the *Kebele*. Although the main focus was on including the SSI implementation *Kebeles* in the sample frame, the AEZs were also considered in the selection process to have a variety of AEZ perspectives. To fill out the questionnaire, experienced survey enumerators who speak the local language (Afan Oromo) and have relevant experiences were recruited and trained. Before the commencement of the household survey, the questionnaire was pre-tested, and the HH survey was conducted during November 2018–February 2019. Table 1 shows the districts' characteristics and the distribution of sample HHs.

**Table 1:** Characteristics of districts and the distribution of sample HHs.

AEZs	Sample Districts	Altitude (m)	Major Livelihood	Major Crop Type	Major Livestock	Number of Respondents		
						IUs	INUs	T
Highland (HL)	Dendi	2300–3600	Mixed farming	Wheat, Barley, Teff	Sheep, Cattle, Equines	66	66	132
Midland (ML)	Adea	1500–2300	Mixed farming	Teff, Wheat, Maize	Cattle, Sheep	66	66	132
Lowland (LL)	Fentale	500–1500	Agropastoralism and Pastoralism	Sorghum, Maize	Camels, Goats, Chickens	66	66	132

m = meter; IUs = irrigation users; INUs = irrigation non-users; T = total.

#### 2.2.2 Data type and sources

The current study is based on a household survey conducted among smallholders in the three districts mentioned above. Key Informant Interviews (KIIs) with selected irrigation users and non-user farmers were conducted to supplement the information of the HH survey. Farmers' livelihoods were characterized using data from the HH survey using indicators that attribute the human, physical, natural, financial, and social capitals (Table 2).

**Table 2:** Livelihood capitals, their indicators, and literature sources.

Livelihood capitals	Indicators	Variable label	Source
Human	Food varieties	Average number of varieties of food consumed by the household in a day	[41]
	Health expenditure	Estimated household's annual health expenditure (ETB)	[52]
	Education expenditure	Estimated household's annual education expenditure (ETB)	[52]
Physical	Crop types	Number of crop types produced in the production year	[26]
	Productive assets	The estimated monetary value of the household's productive assets (ETB)	[68]
	Household assets	The estimated monetary value of the household assets (ETB)	[69]
	Livestock	Number of livestock in the household (TLU)	[70]
Natural	Land expenditure	Estimated expenditure of the household for rented agricultural land (ETB)	[71]
Financial	Saving	The annual amount of money saved by the household (ETB)	[72]
	Credit	The annual amount of credit money accessed by the household (ETB)	[73]
	On-farm income	Annual on-farm income of the household (ETB)	[74]
	Off-farm income	Annual off-farm income of the household (ETB)	[75]
	Non-farm income	Annual non-farm income of the household (ETB)	[76]
	Agricultural input	The annual amount of money spent on agricultural input expenditure (ETB)	[77]
Social	Mobile cellphone	The estimated monetary value of the mobile cellphones in the household (ETB)	[78]
	‘*Idir*’ expenditure	The annual amount of money spent for ‘*idir*’ expenditure (ETB)	[79]

TLU = tropical livestock unit (A TLU is equivalent to 250 kg of live weight of the animal); ETB = Ethiopian Birr; *Idir* = an association of people whose objective is to offer social and financial protection to its members in the events of death.

In studying the impact of small-scale irrigation on the livelihood improvement of farmers, the variables of livelihood indicators in Table 2 were prioritized. In other words, the subject of inquiry was what happened to the livelihood capitals of farmers who used small-scale irrigation compared to their non-user counterparts. Livelihood is conceptualized as a means of living [66] for farmers and was expressed in the five capitals (human, physical, natural, financial, and social) [67]. Significant indicators attributing to these livelihood capitals were carefully selected from the literature and used accordingly. The indicators used to characterize livelihood capitals and their sources are presented in Table 2.

### 2.3 Analytical model

Using small-scale irrigation agriculture impacts rural livelihoods [80,81]. In analyzing the effects of SSI on household livelihoods, it is essential to assume that farm households are risk-neutral. Their decision on whether to practice or not small-scale irrigation agriculture depends on the value of the expected utility of wealth (livelihood) from usage and non-usage [52]. Since livelihood is too broad and complex to capture in a single indicator, selecting certain indicator variables is necessary. Hence, livelihood was considered a function of the five capital assets. Subsequently, as indicated in Table 2, three variables constituting human capital, four variables attributing physical capital, one variable making natural capital, six variables attributing financial capital, and two variables representing social capital were selected. Then, following Khandker et al. [82], for the expected utility of wealth (livelihood), the general model of the study can be given by Equation (2) as follows:

(2) $$U_{i}\left(HHL\right)=Y_{i}\beta +\eta D_{j}+\varepsilon_{i}i=1,2,3,\ldots n$$

where $U_{i}\left(HHL\right)$ is the expected utility of wealth or household livelihood (HHL) for household i; $Y_{i}$ is the vector of observed explanatory variables; $D_{j}$ is the decision to participate in small-scale irrigation ($D_{j}=1\text{,}$ if farmers participate in small-scale irrigation and $D_{j}=0\text{,}$ otherwise); *η* is the impact of participation in SSI on the expected utility of wealth of household livelihood (variables stated in Table 2); $\varepsilon_{i}$ is an error term with a mean of zero and variance $\delta_{\varepsilon }^{2}$ that apprehends the errors in measurement and unobserved factors that affect the participation decision and its outcomes. If SSI was randomly assigned to farmers, the difference in livelihood capital between users and non-users might be used to determine the causal effect of participation in irrigation on farmers' wealth. However, participation in irrigation is not assigned at random. Instead, it is a procedure of ‘self-selection’ by farmers. Whether a farmer participates in irrigation is determined by socio-economic variables at the household level. Because all sample households in the study area are part of the SSI schemes, irrigation does not depend on water access. Water is equally accessible in the scheme to all farmers, and participating and non-participating in small-scale irrigation are based on the farmers' preferences. Hence, all “Irrigator” and “Non-irrigator” farmers have been selected based on the same condition (having access to water sources, but some do irrigation and others do not).

An average treatment effect (ATE) is the expectation of the treatment effect across all farmers [83]. A problem occurs using non-experimental data (such as the data in the current study) because only either $Y_{i}\left(1\right)$ or $Y_{i}\left(0\right)$ is observed for each farmer i, but not both. Hence, the expected utility of wealth (*HHL*) is not observable, and the choice of participation or non-participation is observable; the (*HHL*) is represented by $U^{*}$ (*HHL*). Subsequently, farm households would participate in irrigation only when the participation's expected benefit $\left(U_{i1}^{*}\left(HHL\right)\right)$ exceeds non-participation's $\left(U_{i0}^{*}\left(HHL\right)\right)$.The main objective is to find the estimated average treatment effect for the treated population (${ATT}_{i}$). The average treatment effect is the difference between the treated and controlled smallholder households, which implies whether $\left(U_{i1}^{*}\left(HHL\right)\right)>\left(U_{i0}^{*}\left(HHL\right)\right)$ due to irrigation usage, where ${HHL}_{i1}$ and ${HHL}_{i0}$ are the differences in the expected utility of wealth using the predefined factors if farmers belong to treated or non-treated groups, respectively.

Since the aim is to determine the SSI impacts on household livelihood capital, the question of ''how to estimate the average treatment effect of the livelihood indices'' must be considered. The treatment's effect on the unit i could be observable as $Y_{i}\left(1\right)-Y_{i}\left(0\right)$, if $Y_{i}\left(1\right)$ and $Y_{i}\left(0\right)$ were observable. We may then use this information to estimate the population's average treatment effect (ATT) for the entire sample N [84]. To determine the average treatment effect on the treated $\left(ATT_{i}\right)$, Equation (3) was used. Various evaluation strategies are available, and an appropriate technique has to be chosen depending on the nature of the study and information availability [41].

(3) $${ATT}_{i}=E\left\{{HHL}_{i1}-{HHL}_{i0}|D_{j}=1,P\left(Y_{i}\right)\right\}=E\left({HHL}_{i1}|D_{j}=1\right)-E\left({HHL}_{i0}|D_{j}=0\right)$$

There are different methods by which impacts can be evaluated. These include randomized selection methods, difference-in-difference, instrumental variable estimation, regression discontinuity design, and PSM methods. They vary in their underlying assumptions concerning how to solve the bias in selection in estimating the intervention treatment effect [85]. Randomized assessments involve a randomly assigned initiative throughout a sample of subjects (community or individuals); treatment and control subjects with similar pre-intervention characteristics progress and are followed over time [86]. A difference-in-difference method can be used in experimental and experimental settings. The regression discontinuity method compares participants and non-participants in a close region around the eligibility cutoff using exogenous intervention criteria (eligibility requirements). Finally, PSM approaches examine treatment effects within participants and the matched non-participant units, using a variety of observed attributes to do so [82]. PSM techniques imply that selection bias is based solely on observed features and cannot account for unseen factors influencing participation.

The PSM method is preferred in this study because there was no baseline data on SSI participants and non-participants in the study area. Households were either purposefully placed or self-selected to participate in small-scale irrigation, and the field data was generated based on a cross-sectional survey.

Using observed features, PSM creates a statistical comparison group based on a model of the probability of participating in the intervention [87]. In PSM, the first step is calculating the propensity score and ensuring that the balancing requirement is met. The balancing characteristic of the propensity score, according to Caliendo and Kopeinig [88], is done via Equation (4):

(4) $${HHL}_{i1},{HHL}_{i0}\;\left(⏊|Y_{i}\right)⟹E\left({HHL}_{i1}|P_{i}=1,X_{i}\right)=E\left({HHL}_{i0}|P_{i}=0,Y_{i}\right)$$

The second phase involves matching propensity scores using selection and outcome equations. To estimate the probability of treatment for each observation and generate the collection of matched observations, the selection equation (5) is used. The binary logit model can estimate the value of the propensity score (selection equation). Because the study's participation choice contains dichotomous values, this is the case (1 for farmers who participated in irrigated farming and 0 otherwise). To do so, one must make a common support assumption. By this assumption, treatment units must be comparable to non-treatment units in terms of observed attributes unaffected by participation; as a result, some non-treatment units have to be dropped to ensure comparability. Comparing incomparable individuals, breaking this assumption is a primary source of bias.

(5) $$P\left(D_{i}=1|X_{i}\right)=\Phi \left(f\left(X_{i}\right)\right)=\sum_{i=1}^{n}a_{i}X_{i}+\varepsilon_{i}=\frac{e^{f\left(X_{i}\right)}}{1+e^{f\left(X_{i}\right)}}⟹\hat{P}\left(X_{i}|D\right)=1$$

where Φ denotes the normal cumulative distribution of the livelihood index's function and $f\left(X_{i}\right)$ represents a specification of the household practicing irrigation. A propensity score will be calculated for each participant and non-participant household, which might be a continuous variable with a value between 0 and 1.

Lastly, suppose the propensity score model is statistically significant; SSI participants and non-participants will be matched using criteria, including Nearest-Neighbor matching, Radius matching, Kernel matching, Mahalanobis matching, and Stratification matching [89]. The weights assigned by the matching process may impact the resultant intervention estimate. It is advisable to use and compare all the matching criteria [90].

## 3 Results and discussions

### 3.1 The socio-economic and demographic characteristics of the households

Table 3 depicts the socio-economic and demographic characteristics of the study households, disaggregated by irrigation users and non-users. Relatively, irrigators are younger than non-irrigators. The mean age of the households for irrigators and non-irrigators was 40.5 and 41.6 years, respectively. Similarly, the average family size of irrigators was 4.26, larger than that of non-irrigators. Since irrigation is more labor-demanding activity, the younger age of the irrigation users explains this truth.

**Table 3:** Socio-economic and demographic characteristics of the study households (ANOVA for mean and Chi-Square Test for percent).

Variables	Irrigators	Non-irrigators	Average	*P*-value
AGE-HH	40.5	41.6	40.8	0.210
MH-HH	85.9%	83.8%	84.8%	0.337
SIZE-HH	4.26	3.98	4.12	0.102
MR-HH	85.4%	88.9%	87.1%	0.349
LIT-HH	78.3%	75%	76.6	0.238
AGRI-HH	86.8%	85.3%	86%	0.544
LAND-HH	1.61	1.39	1.50	0.165

ANOVA = analysis of variance; AGE-HH = age of the household head (years); MH-HH = male-headed household; SIZE-HH = household size; MR-HH = married household head; LIT-HH = literate household head; AGR-HH = households with agriculture their primary occupation; LAND-HH = Landholding size of the households in a hectare.

Similarly, the diversity in family size between irrigators and non-irrigators can be accounted for by the level of labor required for irrigation practices. Moreover, irrigation users and non-users were diverse in terms of the education level of the study household head. Accordingly, 78.3% of the irrigation users and 75% of the non-users were literate, indicating irrigation users' more literate status. Agriculture as the main occupation was 86.8% and 85.3% for irrigation users and non-users, respectively.

The One-way ANOVA and Chi-Square independent tests showed statistically non-significant differences between irrigation users and non-users across all the socio-economic and demographic variables. Landholding size between irrigators and non-irrigators was computed. On average, irrigators possess 1.61 ha of land, while non-irrigators possess 1.39 ha of land. As can be seen from Table 3, the One-way ANOVA result indicated that there is no statistically significant land holding size between the two groups. This result suggests that the allocation of households in the treated and control group was based on a randomized control trial. In other words, households were randomly assigned to the treated and control groups to determine the impact of small-scale irrigation on livelihood improvements, which contributed positively to the robust performance of the PSM model. This is because, except for the treatment, random assignment ensures that the only difference in attributes between the two groups is entire to treatment (small-scale irrigation intervention). As a result, the difference in outcomes following the intervention should be attributed to the treatment, which shows the treatment's causal influence [91].

### 3.2 Description of the explanatory variables

**Fig. 2:**
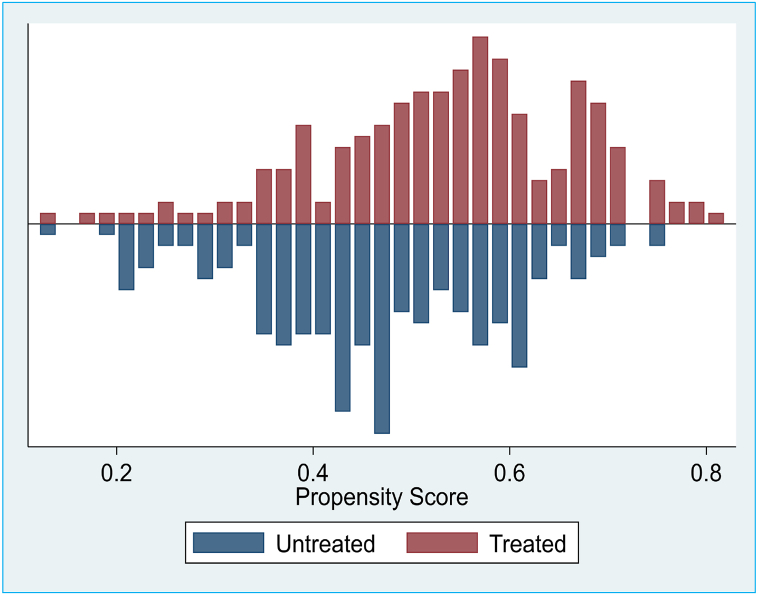
Balance for propensity score before matching.

As illustrated in Fig. 2, most covariates were similar between the treated and untreated groups during the pre-matching stage. After the matching process, more similarity was observed in the covariates for the treated and untreated groups ensuring the selection of PSM to compare the two groups was appropriate and precise. The balancing property of the propensity score was found to be satisfactory, and the assumptions made were met.

Before doing the propensity score, some explanatory (dependent) variables were used to estimate the mean of a continuous dependent variable. As Table 4 depicts, four of the selected explanatory variables (proportion of male-headed households, literate household heads, perceived food security status, and size of agricultural land) were statistically significant at different levels. This suggests that these variables were the most critical determinants in explaining the likelihood of farmers participating in small-scale irrigation.

**Table 4:** Estimation of the coefficients of the propensity score in the logit regression model.

Variables	Coefficients (Standard error)	Variables	Coefficients (Standard error)
AGE-HH	0.004 (0.010)	LIT-HH	−0.017 (0.009)**
M-HH	−0.344 (0.207)*	PER-FSS	−0.305 (0.148)**
SIZE-HH	−0.011 (0.043)	ACC-FC	−0.100 (0.147)
MR-HH	0.103 (0.075)	AGRI-LAND	−0.266 (0.072) ***
cons	1.392 (0.482) ***	LR-HH	−0.008 (0.009)
Obs. = 396; LR Chi2 (9) = 28.98; Prob > Chi2 = 0.0007; Log Likelihood = −259.9975			

∗∗∗Significant at p < 0.01; ∗∗significant at p < 0.05; ∗significant at p < 0.1.

PER-FSS = perceived food security status; ACC-FC = access to financial credit; AGRI-LAND = size of agriculture land (ha); LR-HH = length of residence of the household head (years); Obs. = observations; LR = logistic regression.

### 3.3 Impact of small-scale irrigation on farmers' livelihood improvement

To investigate the impact of practicing SSI on livelihood, we have attributed livelihood in the five capitals (human, physical, natural, financial, and social) and identified relevant indicators as demonstrated in Table 2. Table 5, Table 6 show the PSM's average treatment effects in the treatment (SSI users) and control groups (SSI non-users). Five matching methods (Nearest Neighbor, Radius, Kernel, Mahalanobis, and Stratification) were used. Diaz and Handa [89] suggested using all available matching criteria to examine the differences between each technique. The following sections illustrate these impacts categorized by the five livelihood capitals.

**Table 5:** Average treatment effect (standard errors in brackets) for human, physical, and natural capital variables.

Livelihood capital	Livelihood Indices	Nearest Neighbor Matching	Radius Matching	Kernel Matching	Mahalanobis Matching	Stratification Matching
	VFC	0.28 (0.13)**	0.18 (0.11)*	0.22 (0.10)**	0.31 (0.12)*	0.25 (0.11)**
Human	EDUCEXP	728.83 (438.00)*	−356.38 (514.09)	−5.47 (520.45)	288.57 (565.96)	−28.44 (513.72)
	HLEXP	173.99 (215.93)	−85.05 (367.28)	−135.65 (340.75)	317.10 (186.92)*	−175.89 (411.76)
	CTYP	0.60 (0.17)***	0.60 (0.15)***	0.57 (0.14)***	0.56 (0.16)***	0.59 (0.14)***
Physical	PRASSET	398.04 (251.11)	391.28 (263.15)	198.68 (249.10)	237.70 (292.95)	233.65 (291.37)
	HHASSET	5438.34 (2815.84)*	4513.30 (2869.80)	4374.63 (2729.84)	5598.41 (3217.25)*	3932.19 (2370.64)*
	LVST	0.90 (1.11)	0.56 (1.01)	0.94 (0.96)	1.24 (1.06)	0.78 (1.01)
Natural	LANEXP	3064.77 (709.51)***	2953.19 (791.93)***	2877.91 (755.96)***	3118.24 (877.12)***	2821.33 (677.87)***

∗∗∗Significant at p < 0.01; ∗∗significant at p < 0.05; ∗significant at p < 0.1.

VFC = variety of food consumed in 24 h; EDUCEXP = education expenditure (ETB); HLEXP = health expenditure (ETB); CTYP = crop types produced in the past production year; PRASSET = monetary value of productive assets (ETB); HHASSET = monetary value of household assets (ETB); LVST = number of livestock owned by the household (tropical livestock unit); LANEXP = agricultural land rent expenditure (ETB).

**Table 6:** Average treatment effect (standard errors in brackets) for financial and social capital variables.

Livelihood capital	Livelihood Indices	Nearest Neighbor Matching	Radius Matching	Kernel Matching	Mahalanobis Matching	Stratification Matching
	SAVE	2833.01 (1867.03)	3187.41 (1517.11)**	3076.85 (1456.60)**	3601.65 (1819.99)*	2844.87 (1517.31)**
	CREDIT	−2927.98 (1449.80)**	−2744.72 (880.53)***	−2440.55 (837.18)***	−1613.59 (1427.41)	−1478.48 (1048.14)
Financial	ONFINC	7730.62 (2358.28)***	8176.91 (2069.67)***	8014.28 (2010.05)***	9024.48 (2267.63)***	8133.09 (2016.72)***
	OFFINC	1737.57 (487.48)***	776.25 (725.20)	519.15 (696.93)	−194.10 (868.39)	513.20 (669.58)
	NFINC	3727.46 (1598.71)**	3766.27 (1466.77)**	3509.47 (1426.99)**	2734.66 (1735.43)	3395.27 (1459.54)**
	INPEXP	3718.32 (977.98)***	3268.03 (1034.05)***	3230.31 (998.87)***	3688.97 (983.31)***	3192.16 (942.26)***
Social	MOBCEL	496.79 (215.77)**	400.61 (207.84)*	405.06 (199.30)**	529.67 (213.82)**	410.46 (190.12) **
	EDREXP	55.87 (61.26)	55.61 (57.98)	57.37 (55.62)	57.83 (58.28)	60.07 (51.75)

∗∗∗Significant at p < 0.01; ∗∗significant at p < 0.05; ∗significant at p < 0.1.

SAVE = amount of money saved by the household in a year (ETB); CREDIT = amount of annual credit accessed by the household for production purposes (ETB); ONFINC = annual on-farm income (ETB); OFFINC = annual off-farm income (ETB); NFINC = annual non-farm income (ETB); INPEXP = annual agricultural input expenditure (ETB); MOBCEL = monetary value of mobile cell phones in the household (ETB); EDEXP = annual *idir* expenditure (ETB).

#### 3.3.1 Human capital assets

Human capital was attributed to three variables: the variety of food consumed by the household members in a day and the health and education expenditures of the household. As can be seen from Table 5, participation in SSI in the upper Awash sub-basin had a positive and statistically significant effect on the variety of food consumed by the household at the 95% level at the Nearest Neighbor, Kernel, and Stratification matching criteria; and at 90% using the Radius and Mahalanobis methods. For instance, participation in SSI increased the variety of household food consumed by 0.28 ± 0.13 and 0.31 ± 0.12 based on the Nearest Neighbor and Mahalanobis methods at 95% and 90% significance levels, respectively.

A balanced diet is an essential aspect of human development. One of the irrigator farmers who took part in the key informant interview (KII) from the Adea district stated that one of the main benefits of being an irrigator is having fresh and diverse food on the plate throughout the year. Farmers ascribe this to the opportunity to produce all over the year. In this relation, Passarelli et al. [32] reported that using small-scale irrigation enhances the consumption of a balanced diet by diversifying farmers' production and leading farmers to higher agricultural income. The result also suggests that irrigation is vital adaptation practice and helps meet critical sustainable development goals [27]. Differences were observed regarding education and health expenditures between the SSI users and non-users. Participation in SSI increased the households' annual education and health expenditures by 728 ± 438 SE and 317 ± 186 SE ETB, respectively, as per the Nearest Neighbor and Mahalanobis methods results and at a 90% significance level (Table 5). However, the differences were not statistically significant for most matching methods. Rural areas are known for free access to public education and health service centers, reducing the differences in the expenditures between irrigators and non-irrigators. Zeweld et al. [52] have also found statistically non-significant education expenditure differences between SSI adopters and non-adopters in the rural areas of northern Ethiopia. To generalize, participation in SSI has positively contributed to the human capital assets enhancement of rural households in the upper Awash sub-basin.

#### 3.3.2 Physical capital assets

The study found statistically significant differences in physical capital elements, such as the type of crops produced and the monetary value of household assets, at 99% and 90%, respectively (Table 5). Variables such as the type of crops grown, the value of productive and household assets (e.g., irrigation watering jar, spade, sickle, etc.), and ownership of livestock (in TLU) characterized the physical capital of the study households (see also [92,93]. The differences in the monetary value of productive assets and livestock ownership between the SSI participant and non-participant groups were statistically non-significant. The non-significant difference in TLU can be explained by the fact that farmers use common grazing areas for livestock grazing. Despite the difference between the two study groups, the issues of common property resources might have influenced the result. One of the roles of SSI utilization is producing verities of the crop. That was why the difference between the two groups of farmers was highly significant for all five estimation methods. The discussion we had with KII also substantiates these findings. In this regard, KII participants from the Dendi district explained that producing varieties of crops would help stabilize household income: when the income from the sale of onions drops due to the market, the price of tomatoes will rise. For example, participation in SSI made households have more than 0.56 ± 0.16 SE to 0.60 ± 0.17 SE varieties of crops produced in a year compared to non-participating counterparts.

Similarly, households participating in SSI possessed more valuable household assets than SSI non-participants. Table 5 shows that irrigators and non-irrigators have a monetary value of household assets differential of 3932 ± 2370 SE to 5598 ± 3217SE ETB, making the earlier groups more advantageous.

#### 3.3.3 Natural capital assets

Land is an essential resource in agriculture. In most places in the upper Awash sub-basin, the availability of agricultural land, either by possession or rent, determines the participation of smallholders in SSI schemes. The suitability of the land for irrigation is also an essential factor. As presented in Table 5, all five estimator models predicted that the difference in expenditure for agricultural land between irrigators and non-irrigators was statistically significant at the 99% level. The result also suggests that the irrigation users' annual land expenditure for the irrigation purpose is more sign up to 3118 ± 877 SE ETB compared to the non-irrigation users. According to interview data, the yearly cost of renting a quarter hectare of irrigation land in some study areas, such as Adea, was up to 5000 ETB. A farmer needs at least 20000 ETB to rent a hectare of land for a production year, which is a very high investment for a smallholder farmer. Due to the higher rent cost, irrigator farmers are more likely to access an additional plot of land to expand their irrigation than non-irrigator farmers. In this case, it is reasonable to infer that the irrigator's expenditure on renting irrigation land has improved due to their participation in small-scale irrigation.

#### 3.3.4 Financial capital assets

Irrigation agriculture requires financial capital to purchase specific irrigation equipment, improved seeds, and agricultural inputs like chemical fertilizers, pesticides, and herbicides [94]. At the same time, participation in irrigation contributes positively to acquiring financial capital assets. As can be referred from Table 6, a statistically significant difference in the annual on-farm income between the irrigator and non-irrigator groups was found at a 99% significance level with all the five matching criteria. The annual non-farm income also showed a statistically significant difference between the two groups at a 95% significance level using the four estimators. It was found that the yearly on-farm income of irrigators was higher (9024 ± 2267 SE ETB), and the non-farm annual income difference between the two groups was estimated at 3766 ETB. Participating in small-scale irrigation agriculture in the study area gave the irrigator group more on-farm and non-farm incomes than the non-irrigator group. The better non-farm income for irrigators was due to the productive assets they acquired besides the irrigation practices. Compared to the non-irrigators, the irrigators have vehicles, mills, and other businesses. The off-farm income was non-significant between the two groups using the four estimators. This implies that irrigation participant households are more likely to purchase agricultural inputs to enhance production. This can be inferred from the agricultural input expenditure findings of this study. There was a significant difference in expenditure on agricultural inputs such as fertilizers, improved seeds, pesticides, and herbicides between irrigation users and non-users at a 99% significance level across all five estimators (Table 6). For example, from the nearest neighbor matching, irrigation users' annual agricultural input expenditure was more significant in 3718 ETB than the non-irrigation users. A further explanation for this is that farmers are risk-averse. Having access to water throughout the year encourages irrigators to invest more in inputs as there is less risk of crop failure with rainfall variability.

Accessing credit money from formal finance institutions aiming for long-term agricultural and non-agricultural investments is almost none for households. As revealed in Table 6, the amount of annual credit accessed showed a significant negative difference between irrigator and non-irrigator at 99%, with Radius matching at 95% levels using Nearest Neighbor and Kernel matchings. This result informed that the non-irrigators accessed more money than the irrigators. The credit is primarily accessed in the study area for daily needs from local individuals and informal village sources. An interviewed small-scale irrigation non-user farmer in the Dendi district also noted, “*I cannot access credit from the banks and formal financial sources as I do not have the collateral it demands. Even if I do, I do not want to have it from these sources as I cannot engage in long-term investments besides farming*”.

In most cases, the irrigators are lenders as they have relatively better annual income. Hussain and Thapa [95] indicated that the probability of smallholders accessing credit from formal financial institutions is limited, especially in developing countries. The above findings suggest that small-scale irrigation participation's financial capital asset impact is the highest of all the remaining capital assets, positively influencing the other capital assets.

#### 3.3.5 Social capital assets

Social capital includes reciprocity, relations of trust, everyday standards, norms, consequences, and connections in institutions. In rural areas, healthy communications and participation in different gatherings define the social capital asset of the community [96]. That was why we used the monetary value of mobile cellphones in the household and the local gathering called ‘*idir*’ to constituent social capital assets in the current study. As presented in Table 6, participating in SSI in the study area had a positive and statistically significant impact on the better monetary value of mobile cellphones at the 95% level by all the estimators except the Radius matching. For instance, using the Mahalanobis matching method, the monetary value of mobile cellphones for the irrigation user group was about 529 ± 213 SE ETB higher than that of the irrigation non-user group. There was no significant difference in the annual ‘*idir*’ expenditure. Although some households have more ‘*idir*’ and hence payments than others, this could be because every household pays the same amount. Maru et al. [26] indicated that the involvement of brokers in marketing irrigation products had been a significant challenge for irrigators. This is due to irrigators' lack of trusted market information and the perishable nature of the products. It has created a distorted market value chain by over-strengthening the role of brokers in the market.

Livelihood capital transformation is more important for rural households to improve livelihood capacities and reduce vulnerabilities [97]. The transformation of one livelihood capital of rural households can also enhance the other livelihood capitals. As shown in Table 6, the financial capital of irrigators is significantly better off than the non-irrigators. This might influence the other livelihood capitals through capital transformation. The higher financial capital gain gave irrigator households better other livelihood capital. For instance, better finance could lead to better human capital for food, education, and health expenditures. The same applies to physical capital in crop production and asset ownership and natural capital such as land expenditure. The current study's findings indicate that the strong impact of participating in small-scale irrigation has boosted the irrigator households' financial capital, influencing the other livelihood capital of households through livelihood capital transformation.

#### 3.3.6 Poverty reduction

Smallholder farmers in Ethiopia account for a large share of the population living in poverty, attributed to low agricultural production [98]. According to Alemu and Singh [99], 39% of the country's population lives in absolute poverty, defined as a daily income of less than 1.25 United States Dollars (USD) per day. Some studies exaggerate the problem, claiming that 90% of the population is impoverished based on multidimensional poverty measures [100].

**Fig. 3:**
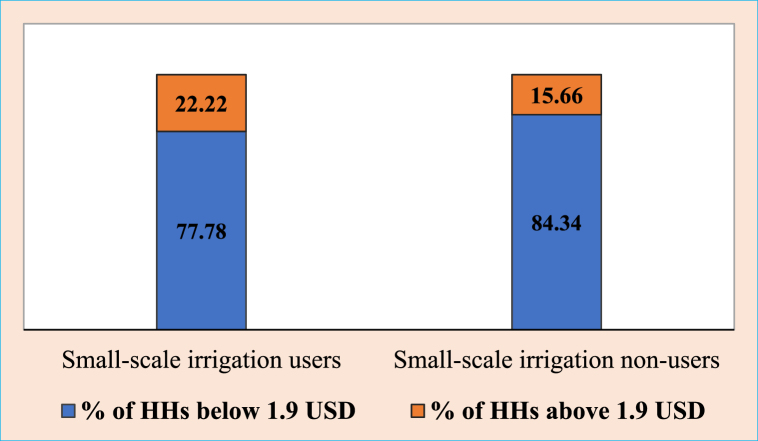
The proportion of households above and below the absolute poverty line.

The World Bank's international poverty line (IPL) of USD 1.90 per day in 2011 purchasing power parities was used to cut the absolute poverty line [101]. The annual income of households from on-farm, off-farm and non-farm sources were aggregated and converted into daily income. The exchange was based on the rate during the field survey period, i.e., from November 2018 to February 2019 (1 USD = 28.1811 ETB). As shown in Fig. 3, most households in the study area were under the IPL. About 77.78% of households from irrigation users and 84.34% of non-irrigation users were below the absolute poverty line. According to the figures, though there was no significant difference between small-scale irrigation users and non-user households, the proportion of non-irrigator households under IPL was more than that of the irrigators. Although income is a substantial determinant of poverty, it is insufficient as a measure [102].

Many poor households in developing countries' rural areas rely heavily on their productive activities for survival and earn very little income [103]. People may also be poor due to structural factors, such as a lack of resources and chances to build a sustainable livelihood and the constant battle to fulfill basic needs [104]. An interviewed irrigation non-user farmer in the Fentale district stated, “*because my family does not participate in small-scale irrigation, we miss out on many opportunities. My neighbor who participates in irrigation works all the seasons and sells some of his irrigation products to* support *his family's income. He has a better quality of life than me*”. Although participation in small-scale irrigation might not significantly support households out of absolute poverty in terms of daily income, it can contribute to users' quality of life more than non-users. However, suppose farmers' current usage of SSI is supported by sufficient irrigation water during all the crop growing periods, appropriate and enough agricultural inputs, and a stable market for irrigation products. In this case, SSI will help smallholders out of poverty. For SSI to assist households out of poverty, it needs to be implemented with better standards [105], starting from ensuring water availability at schemes to the appropriate market facility for the products [106].

## 4 Conclusions

The study analyzed the impact of small-scale irrigation on farm households' five livelihood capital assets in the upper Awash sub-basin, Ethiopia. The study used propensity score matching between treated (irrigators) and control (non-irrigators) to achieve the objective. Nearest Neighbor, Radius, Kernel, Mahalanobis, and Stratification matching models were applied to estimate the magnitude of the impact differences.

The study results show that participation in small-scale irrigation improves most of the indicators considered in the five capital assets. However, the magnitude of the impact and the level of significance varies. For example, for human capital indicators, only the variety of food consumed by the household was statistically significant, which has implications for enhancing the quality of life of irrigation participants. Regarding physical capital assets, a significant difference was observed between irrigators and non-irrigators in the number of crop types produced in the production year. This has increased the stability of farmers' income during fluctuating market prices. In terms of natural capital assets, using all five matching propensity scores, the farmland rental expenditure between the two groups was significantly different at 99%.

Participation in SSI improves farmers' social capital assets. The significant impact of SSI participation was on the financial capital assets. For example, under all five PSM estimates, the annual farm income and expenditure of agricultural inputs at a significance level of 99% show a significant difference between irrigation participants and non-participants. For example, under all five PSM estimates, the annual farm income and expenditure of agricultural inputs at a significance level of 99% show a significant difference between irrigation participants and non-participants. However, considerable market information gaps and the involvement of local brokers in marketing irrigation products have reduced the benefits of irrigation agriculture. Hence, establishing farmers' cooperatives, involving farmers actively in the market value chain, and limiting the involvement of brokers through enforcement of regulations could be the option to manage market distortion affecting farmers.

It is found that participation in SSI has improved most elements of the livelihood capital. Hence, participating the non-irrigator farmers in the irrigation schemes could enhance the livelihood of the non-irrigators. This could lead to a higher demand for the existing irrigation water supply and may result in the poor performance of the overall SSI scheme. Therefore, focusing on considerations such as improving the water usage mechanism and productivity, establishing proper water allocation institutions between up and down streams, and limiting the role of brokers in the irrigation product marketing chain through legal regulations could be future policy directions.

## 5 Limitations of the study

Finding a perfect study design in any scientific assessment is difficult. This study also has its limitations. Besides the utilization of small-scale irrigation, the biophysical and environmental characteristics such as agroecology, access to land, climate difference, crop choice, finance to invest in irrigation, and soil in each sample district can affect the differences in the livelihood capital assets of the smallholder farmers. Hence, the current study did not consider those characteristics directly. Future assessments in the upper Awash sub-basin can bridge this gap by applying appropriate research design to consider those limitations.

## Author contribution statement

Husen Maru: Conceived and designed the study; Performed the experiment; Analyzed and interpreted the data; Contributed reagents, materials, analysis tools or data; Wrote the paper.

Amare Haileslassie and Tesfaye Zeleke: Analyzed and interpreted the data; Contributed reagents, materials, analysis tools or data; Wrote the paper.

## Data availability statement

Data will be made available on request.

## Additional information

No additional information is available for this paper.

## Declaration of competing interest

The authors declare that they have no known competing financial interests or personal relationships that could have appeared to influence the work reported in this paper.
